# Double-bundle ACL reconstruction: influence of femoral tunnel orientation in knee laxity analysed with a navigation system – an in-vitro biomechanical study

**DOI:** 10.1186/1471-2474-9-25

**Published:** 2008-02-25

**Authors:** Stefano Zaffagnini, Danilo Bruni, Sandra Martelli, Naoaki Imakiire, Maurilio Marcacci, Alessandro Russo

**Affiliations:** 1 Laboratorio di Biomeccanica, Istituti Ortopedici Rizzoli, Bologna, Italy; 2 Department of Orthopaedic Surgery, Kyorin University School of Medicine, Kyorin, Japan

## Abstract

**Background:**

This paper reports an in-vitro study for evaluating the influence of the femoral tunnel orientation in anterior cruciate ligament (ACL) double-bundle reconstructions.

**Methods:**

This work describes the experimental protocol and results obtained for six cadaver knees using the FlashPoint optical system (Image Guided, Boulder, Colorado, USA) and a computer-assisted technique for the elaboration of anatomical and kinematic data. Each specimen was examined by the same surgeon in the following steps: (1) intact knee stability was evaluated by performing antero-posterior displacement and internal-external rotation test at 90°; (2) the ACL was resected and the knee evaluated again; (3) the ACL was reconstructed using the gracilis semi-tendinous tendon (through horizontal tunnels in femur), and the new kinematics recorded; (4) the ACL was reconstructed again with the same tendon, but with a more vertical orientation of the femoral tunnel (vertical tunnel) and kinematics was once more recorded; (5) finally the knee was dissected to digitise the anatomical structures.

**Results:**

Off-line computer analysis of the acquired anatomical and kinematic data showed that there was a significant statistical difference (Wilcoxon test with the Montecarlo method for small samples – p = 0.035) between horizontal tunnel (HT) and vertical tunnel (VT) reconstruction both in the antero-posterior test (median antero-posterior displacement in horizontal tunnel was 0.8 mm less than in vertical tunnel reconstruction) and in the internal-external (IE) rotation test (median internal-external rotation in horizontal tunnel reconstruction was 5° less than in vertical tunnel reconstruction).

**Conclusion:**

The analysis of graft behavior in reconstructed knees compared with normal and ACL-deficient knees suggests that the most horizontal tunnel performed better than the vertical tunnel, thus constraining optimally both antero-posterior and internal-external rotations. This finding suggests that femoral tunnel direction may be an important issue in ACL surgery.

## Background

Anterior cruciate ligament (ACL) reconstructions are one of the most frequent operations in athletes. Although the results are certainly satisfactory, in the literature there is still unfavourable outcomes up to 10% [1].

With the aim of improving the final results, recently many surgeons have started performing a double-bundle (DB) reconstruction, thus trying to restore the normal ACL function. Infact, several research studies have shown the different behavior of antero-medial (AM) and postero-lateral (PL) bundles of the ACL regarding orientation [2], force distribution [1], and tension during range of motion [3]. Moreover, Zantop [4] has recently shown that the kinematics expecially for the anterior tibial translation in low flexion angles under Lachman test and simulated pivot shift test can be contributed to the integrity of the PL bundle. According to several authors, anatomic double-bundle reconstruction should reproduce more closely the anatomy and biomechanics of the native ACL. For example Yagi [5] recently investigated the biomechanical effect of anatomic double bundle reconstruction with autologous implants with respect to single bundles, while Guardamagna [6] evaluated in a cadaver study the effect of AM and PL bundles reconstructed with synthetic implants. These studies underlined that double bundle reconstructions more closely reproduce the functional behavior of the normal ACL. Double bundle techniques present many surgical variables and Petersen [7] has shown that a second tibial tunnel provides better pivot shift control compared to two femoral tunnel and one tibial tunnel ACL reconstruction. On the other hand, previous studies emphasized the importance of femoral tunnel orientation to control anterior tibial translation and rotatory laxities [8,9]. Recently, Yamamoto [1] also reported a good control of rotatory laxities when a lateral femoral tunnel was performed in single bundle reconstruction. All theses recent studies underline the importance of femoral tunnel orientation for better performances of ACL reconstruction. This topic however has not been completely addressed in double bundle reconstruction.

The purpose of this study was to analyze the effect of two different femoral tunnels orientation in a double bundle reconstruction, while maintaining the same femoral insertion. The hypothesis was that also in double bundle reconstruction the orientation of femoral tunnel could affect the final knee stability. In particular, we evaluated their influence on final knee antero-posterior and internal-external stability using a system dedicated to computer-assisted surgery, able to provide an accurate evaluation of anatomy and kinematics.

## Methods

### Materials

Six intact fresh-frozen human cadaver legs were examined. Donors were aged between 21 and 53 years old, 2 females and 4 males, 3 knees were left and 3 were right. All knees showed normal anatomy and a normal passive range of motion. Specimens were stored at -20°C from the time of retrieval and were thawed overnight at room temperature prior to testing, thereby undergoing a single freeze cycle. In each specimen the complete femur, the tibia and the foot were maintained intact during the experiment. The skin and muscles surrounding the femur, more than 20 cm from the joint line, were removed so that the bone was exposed for better fixation to the experimental set up. The specimens were kept moist during preparation and testing, by spraying with 0.9% normal saline solution. The anatomical and kinematic acquisitions were performed using FlashPoint optical navigation system (Image Guided, Boulder, Colorado, USA) to accurately record the relative motion of the tibia and the femur and to digitize anatomical data [10,11].

### Experimental procedure

**Figure 1:**
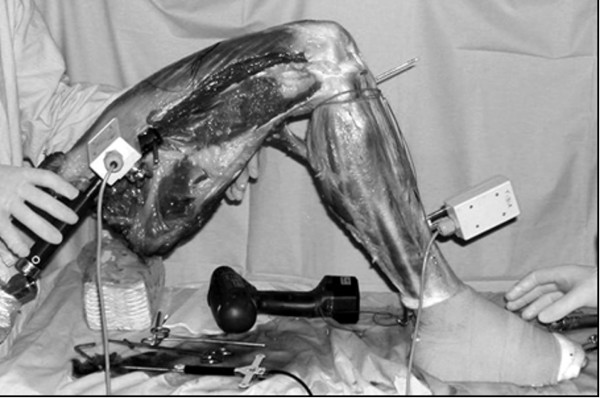
Experimental Set-up.

The femur was fixed to the experimental desktop with a clamping device at 90° of flexion, by suitable supports at the thigh. The tibia was left free to move as in the standard operating room set-up. The intact foot enabled the surgeon to check the internal-external alignment of the limb and use mobile supports at the foot to stabilize the leg better during laxity tests in flexion (Fig. 1).

Two custom rigid bodies supporting a set of standard FlashPoint infrared emitters were fixed respectively to the femur and tibia, to record their relative position during passive motion tests. In order to minimize possible interactions with surgical acts, the femoral rigid body was fixed in the proximal part of the femur and the tibial one distally in the medial part of the tibia (Fig. 1). The accuracy of the system evaluated in vitro is 0,3 mm and 0,3° [12].

First, the intact knee was examined by kinematic tests simulating the standard clinical examination used to diagnose ACL deficiency. In particular, we acquired anterior-posterior drawer tests and internal-external rotations at 90° of knee flexion performed manually at maximum force. This has shown to be quite repeatable in literature [12]. All tests were repeated three times by the same surgeon. We decided to not perform the test at 30° because the mainteinance of the position during tests could be difficult and the gravity of the specimens as well as the meniscal and cartilagineous conditions of the knees could have in certain way affected the repeatability of our measurements. With our set up we consider that the examination at 90° should be the one more consistent during all tests. These 6 degrees of freedom (DOF) were recorded at 18 Hz with the FlashPoint system. Since all the kinematic data were analyzed off-line, the surgeon was blinded with regards to the result of laxity tests. Then the ACL was transected with a minimal incision and kinematic tests were repeated identically for the ACL-deficient knee.

**Figure 2:**
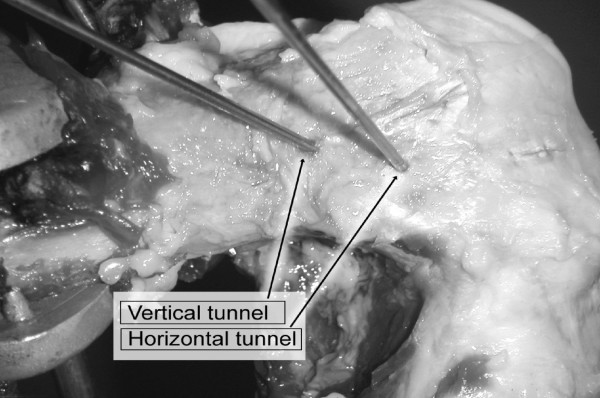
HT and VT tunnels for ACL reconstruction at 90° of flexion.

Subsequently, two ACL reconstructions were performed on the same knee, using previously harvested semitendinosus and gracilis tendons sutured together with the tibial insertion left intact [13]. Both ACL reconstructions used the same single tibial tunnel, drilled 50 mm deep from the tibial spine, medially with respect to the cresta tibialis. The tibial tunnel was directed to the ACL area by outside-in, oriented from medial to lateral in the frontal view and from anterior to posterior in the sagittal view [13]. Two femoral tunnels were made starting from the same area, at the so-called "10:30 hours" position inside the femoral ACL insertion. Femoral tunnels were performed in-out from the antero-medial portal with the knee flexed at 130° using a Kirschner wire and were drilled using a 6-mm diameter cannulated drill (Acufex, Smith&Nephew Inc., Andover, D). For the first femoral tunnel, hereafter "horizontal tunnel" (HT), the surgeon directed the tunnel towards the end of lateral posterior condyle, while for the second femoral tunnel, hereafter "vertical tunnel" (VT), the tunnel was directed 20 mm more proximal with respect to Horizontal Tunnel exit, laterally on the femoral shaft (Fig. 2).

**Figure 3:**
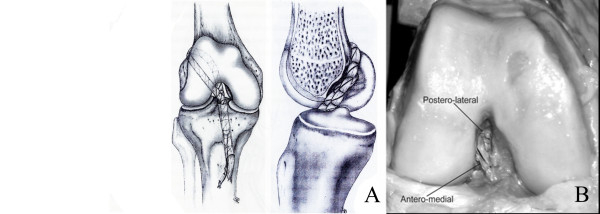
**a and b – ACL reconstruction with the double – bundle technique**. Figure A: descriptive representation. Figure B: reconstruction during experiments.

The graft was pulled through the tibial tunnel and passed into the femoral notch as a postero-lateral bundle by an over-the-top passage performed using a curved Kelly clamp. The graft was then taken from the lateral side of the knee and passed through one femoral tunnel back distally in the joint and into the tibial tunnel again, thus building the antero-medial bundle of the reconstructed ACL (Fig. 3a, 3b).

The procedure was repeated for the second reconstruction passing the graft in the other femoral tunnel. In 3 alternated cases the horizontal tunnel was used first and in 3 alternated cases the vertical tunnel was used first. The prepared graft was preconditioned by moving the knee through 5 cycles of the full range of flexion applying 22 N tension to the graft. Both ACL reconstructions were fixed to the tibia by means of two titanium barbed staples (Citieffe Inc., Bologna, Italy) set at 10° flexion after applying maximum load. The laxity tests were recorded respectively after the horizontal tunnel and vertical tunnel reconstruction. The knee was dissected only after surgery, thus exposing ligaments and bone surfaces in order to digitize accurate data about knee anatomy. A standard joint coordinate reference system (i.e. mechanical axis of femur and tibia, transepicondylar line, joint line and knee center), ACL insertions, tunnel entrance and exit holes, distal femur and proximal tibia surfaces were acquired.

### Analysis

**Figure 4:**
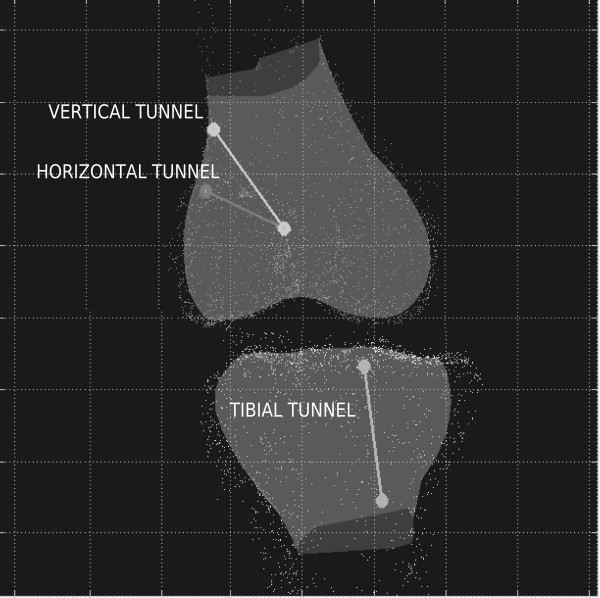
**Computer elaboration of experimental anatomical data of the ACL reconstruction**. In the display reference frame: frontal view at full extension with femoral axes, tibial axes and tunnels evidenced.

Analysis of the knee joint kinematics was performed off-line with a custom dedicated software [14,2,16]. Three-dimensional anatomical data were displayed in a reference system defined on the femur as follows: (1) proximo-distal axis was defined as the tibial mechanical axis in full extension; (2) medio-lateral axis was defined as the transepicondylar line, normalized with respect to (1); antero-posterior axis was defined as the instantaneous cross product of (1) and (2) (Fig. 4). Kinematic data were used to compute the antero-posterior (AP) laxity and the internal-external (IE) laxity of the joint at 90°, using Grood and Suntay decomposition algorithm [17,18].

We also computed the angles described by the two different tunnels in the frontal plane with respect to the femoral mechanical axis and the final length of the graft trajectory in the two reconstructions in extension as the sum of the following values: twice the length of the tibial tunnel, the length of the femoral tunnel, the length of the over-the-top passage of the graft around the posterior condyle, the linear length of the two bundles of the graft replacing the ACL in the femoral notch.

Statistical analysis was performed to assess the precision of the results and the differences between the reconstructions using the two tunnels. In particular, the repeatability of each test was estimated as the percent mean standard error of the three acquisitions of the test on each specimen and a statistical descriptive analysis was made for all results, using the mean values of repeated tests for each specimen.

The global effect of the two reconstructions was estimated by performing the Wilcoxon test with the Montecarlo method for small samples, applied between ACL-deficient knees and horizontal tunnel reconstructions and between ACL-deficient knees and vertical tunnel reconstructions, respectively. Additionally, the functional restoration was evaluated by performing Wilcoxon test with the Montecarlo method for small samples between ACL-intact knee and horizontal tunnel reconstructions and between ACL-intact knee and vertical tunnel reconstructions, respectively. Moreover the Wilcoxon test with the Montecarlo method for small samples was performed to evaluate the difference between horizontal tunnel reconstruction and vertical tunnel reconstruction.

## Results and Discussion

Analysis of the acquired anatomical data showed that the tibial tunnel was performed in the in the postero-medial part of the native ACL insertion and from anterior to posterior at 26 ± 4° with respect to the mechanical axis of the leg, coinciding with the long tibial axis, in the sagittal view [13]. With respect to the femoral mechanical axis in the frontal plane, the horizontal tunnel was set at 57 ± 10°, while vertical tunnel was oriented at an angle of 36 ± 9° (Fig. 4).

The repeatability of antero-posterior laxity and internal-external laxity tests in our sample data was good, showing a percent mean standard error of 8.8% (0.4 mm) and 3.3% (1°), respectively. Also the directions of antero-posterior and internal-external laxity tests were estimated in the computer elaboration, showing a standard error in repeatability less than 10% (3.00°) [18].

**Figure 5:**
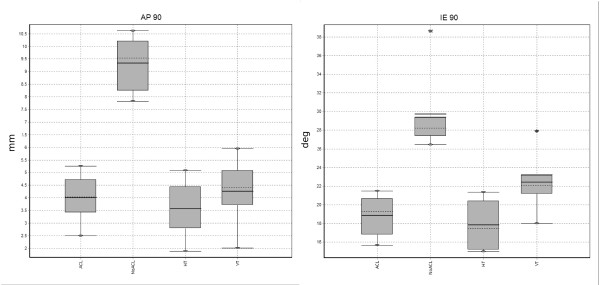
**Boxplot of Knee Laxity in Different Knee Conditions**. Legend: ACL = Knee with Intact ACL Ligament, NoACL = Knee with Resected ALC Ligament, HT = Horizontal Tunnel ACL Reconstruction, VT = Vertical Tunnel ACL Reconstruction.

The antero-posterior laxity and internal-external laxity of the intact knee was significantly increased by ACL resection, as shown by the results of the descriptive comparative statistical analysis (Fig. 5). In particular, the Wilcoxon test with the Montecarlo method for small samples showed significant statistical differences between ACL-deficient and horizontal tunnel reconstruction (p = 0.035) and between ACL-deficient and vertical tunnel reconstruction (p = 0.035), in both antero-posterior and internal-external laxity tests at 90° (See Table 1 and Table 2).

**Table 1:** Wilcoxon Test Evaluated by the Montecarlo Method for Small Samples Applied on AP Stress

**AP 90**			**HT vs NoACL**	**VT vs NoACL**	**HT vs ACL**	**VT vs ACL**	**HT vs VT**
**Z**			-2,201	-2,201	-1,153	-1,153	-2,201
**MonteCarlo 2 Tails**	**p**		0,035	0,035	0,311	0,311	0,035
	**IC 99%**	**Sup**	0,028	0,028	0,293	0,293	0,028
		**Inf**	0,042	0,042	0,330	0,330	0,042

Statistical test applied to compare AP displacements in HT and VT reconstructed knee, with ACL-Deficient (I and II columns), with ACL-Intact (III and IV columns), and among them (V column).

**Table 2:** Wilcoxon Test Evaluated by the Montecarlo Method for Small Samples Applied on IE Stress

**IE 90**			**HT vs NoACL**	**VT vs NoACL**	**HT vs ACL**	**VT vs ACL**	**HT vs VT**
**Z**			-2,201	-2,201	-1,153	-2,201	-2,201
**MonteCarlo 2 Tails**	**p**		0,035	0,035	0,311	0,035	0,035
	**IC 99%**	**Sup**	0,028	0,028	0,293	0,028	0,028
		**Inf**	0,042	0,042	0,330	0,042	0,042

Statistical test applied to compare IE rotations in HT and VT reconstructed knee, with ACL-Deficient (I and II columns), with ACL-Intact (III and IV columns), and among them (V column).

However, a slight difference appeared between the two reconstructions when comparing restored kinematics after the intervention with the intact knee. AP stability after both reconstructions presented statistical homogeneity with the ACL-intact knee according to the Wilcoxon test (p = 0.311), even if horizontal tunnel tended to over-constrain (3.6 mm – median value) and vertical tunnel to under-constrain (4.4 mm – median value) the anterior drawer test with respect to the normal knee (4 mm – median value – See Table 1 and Fig. 5).

On the contrary, internal-external stability after horizontal tunnel reconstruction was homogenous with the intact knee according to the Wilcoxon test (p = 0.311), while vertical tunnel reconstruction presented a minor control of the internal external laxity at 90°, with respect to the ACL-intact knee (See Table 2 and Fig. 5).

The analysis of the differences between the 2 reconstructions confirmed that horizontal tunnel was able to provide a better stability than vertical tunnel; in fact there was a significant statistical difference (p = 0.035) between horizontal tunnel and vertical tunnel reconstruction both in the antero-posterior test (median AP displacement in horizontal tunnel was 0.8 mm less than in vertical tunnel reconstruction) and in the internal-external test (median internal-external rotation in horizontal tunnel reconstruction was 5° less than in vertical tunnel reconstruction).

The mean difference in the lengths of the graft with horizontal tunnel and vertical tunnel was 20 ± 7.9 mm on average. This difference was due to the difference in the two femoral tunnels' length (10.4 ± 3.1 mm) and the differences in the length of the over-the-top wrapping around the posterior condyle (9.7 ± 4.8 mm), as the tibial tunnel and the intra-articular graft bundles were common in both reconstructions.

## Conclusion

In our experiments both reconstructions were able to correct the instability due to ACL rupture in antero-posterior displacements and internal-external rotations and results were comparable with other cadaver studies [19-22]. This study confirms our hypothesis that also in double bundle reconstruction the tunnel orientation affects the final laxity.

The performed reconstruction is a double-bundle reconstruction that involves a single tibial tunnel that is usually performed in the more posterior area of ACL insertion. However, this type of reconstruction, does not completely reproduce the anatomy of ACL: in fact the tibial ACL insertion is not covered entirely, and the postero-lateral bundle, going over the top on the condyle, lacks a femoral insertion [13]. Certainly this procedure has not to be considered as an anatomic reconstruction described by Fu [23] and Yasuda [24], where the PL bundle is really anatomically placed.

However, the results of this experimental setup showed that horizontal femoral tunnels, in this specific reconstruction, can be preferable to the vertical ones. In fact, the horizontal orientation showed an improved overall stability both in antero-posterior and in internal-external laxity tests at 90°. Actually, horizontal tunnel reconstruction seems to over-constrain the knee (although is most similar to the intact knee especially in internal-external rotations) while the vertical tunnel seems to maintain a residual laxity with respect to the normal knee. Certainly Vercillo showed [25] that a fixation of the graft at higher flexion angles may overconstrain the double bundle reconstruction. This could be happened also in our set up.

An additional advantage of horizontal tunnel over vertical tunnel reconstruction is the reduced length of the graft trajectory, which guarantees a good fixation of the harvested tendons to the bone in most cases. The data is also very important for the single bundle reconstruction. A transtibial drilling technique may result in a more vertical orientation whereas the portal drilling results in a more horizontal tunnel orientation. The analysis of the effect of femoral tunnel orientation on a double bundle technique provides a new input, although previous studies have analyzed this aspect in single bundle reconstructions, showing how important is this factor in surgical techniques.

An original aspect of our study is that we did not evaluate the different orientation in relation to different hole positions in the femoral notch, but only the effect of changing the orientation while maintaining the same insertion at "10:30 hours" position. Another important aspect of this study is the use of a computer-assisted methodology, which provided good repeatability and the capability to quantify reliably anatomical and kinematic features.

Our results suggest that the femoral tunnel orientation may affect the final knee behaviour during stress tests, as already described by Woo [8,26] for a single bundle reconstruction, even when the insertion of the graft in the femoral notch remains the same. The effect of femoral tunnel orientation appeared to be significant, especially for rotatory stability as also demonstrated in previous studies comparing an anatomical double bundle reconstruction with a single bundle reconstruction with a different position of the femoral tunnel insertion. Yagi, Yamamoto, Woo and Scopp [8,26,9,5,1] found that a more horizontal position of the graft guarantees better control of rotational instability.

The results of our study suggest that also in double bundle reconstruction a change in tunnel orientation, maintaining the same femoral insertion in the femoral notch, could modify the antero-posterior and rotational stability of our reconstruction. This finding is important not only in our unusual and not completely anatomical double bundle technique, but could be even more important when two femoral tunnels are performed for the reconstruction.

The different kinematic performance of the reconstruction with horizontal tunnel and vertical tunnel may be explained by the different global length of the tendon used, different positions of the postero-medial bundle of ACL reconstructions, although small, or the different force transmission in the transition between intra-articular area and tunnels.

A limitation of this study is that it was performed on a relative small sample and in a cadaver set-up. In-vitro conditions allowed a precise comparison of individual normal, pathological and reconstructed conditions which is difficult in-vivo, but may introduce some biases due to different ligament elasticity of living tissues. Moreover, the tests were performed only at 90° of flexion and the information that could be obtained with test executed also at 30° may have provided more data especially for the posterolateral bundle. No standard load was applied to the tests and only normal maximum manual tests were performed. These factors could have influenced our results especially in cadaver setting. However, the findings of this preliminary cadaver study suggest that this issue could be clinically relevant in changing the final stability of ACL reconstructions, and deserves further analyses, possibly with a similar clinical protocol using a navigation system and a suitable computer-assisted procedure.

## Competing interests

The author(s) declare that they have no competing interests.

## Authors' contributions

SZ conceived the design of this study, participated in the acquisition of data, and in the drawing up of the manuscript. DB contributed in the operative procedures and in the drawing up of the manuscript. SM participated in the design of the study, coordinated the acquisition of data, performed the statistical analysis, and revised the manuscript critically. NI managed the acquisition of data and participated in the drafting of the manuscript. AR contributed in the operative procedures and in the drawing up of the manuscript. MM conceived the design of this study, participated in the acquisition of data, and in the drawing up of the manuscript. All authors read and approved the final manuscript.

## Pre-publication history

The pre-publication history for this paper can be accessed here:



## References

[B1] Yamamoto Y (2004). Knee stability and graft function after anterior cruciate ligament reconstruction. Am J Sports Med.

[B2] Zaffagnini S, Martelli S, Acquaroli F (2004). Computer investigation of ACL orientation during passive range of motion. Comput Biol Med.

[B3] Amis AA (1991). Functional anatomy of the anterior cruciate ligament. Fiber bundle actions related to ligament replacements and injuries. J Bone Joint Surg Br.

[B4] Zantop T, Herbort M, Raschke MJ, Fu FH, Petersen W (2007). The role of the anteromedial and posterolateral bundles of the anterior cruciate ligament in anterior tibial translation and internal rotation. Am J Sports Med.

[B5] Yagi M, Wong EK, Kanamori A, Debski RE, Fu FH, Wo SLY (2002). Biomechanical analysis of anatomic anterior cruciate ligament reconstruction. Am J Sports Med.

[B6] Guardamagna L, Seedhom BB, Ostell AE (2004). Double-band reconstruction of the ACL using a synthetic implant: a cadaveric study of knee laxity. J Orthop Sci.

[B7] Petersen W, Tretow H, Weimann A, Herbort M, Fu FH, Raschke M, Zantop T (2007). Biomechanical evaluation of two techniques for double-bundle anterior cruciate ligament reconstruction: one tibial tunnel versus two tibial tunnels. Am J Sports Med.

[B8] Loh JC, Fukuda Y, Tsuda E, Streadman RJ, Fu FH, Woo SL (2003). Knee stability and graft function following anterior cruciate ligament reconstruction: comparison between 11 o'clock and 10 o'clock femoral tunnel placement. Arthroscopy.

[B9] Scopp JM, Jasper LE, Belkoff SM, Moorman CT (2004). The effect of oblique femoral tunnel placement on rotational constraint of the knee reconstructed using patellar tendon autografts. Arthroscopy.

[B10] Martelli S, Zaffagnini S, Falcioni B, Motta M (2001). Determination of an optimal kinematic protocol for computer-assisted evaluation of anterior cruciate ligament deficiency. Ann Biomed Eng.

[B11] Martelli S, Bignozzi S, Bontempi M, Zaffagnini S, Garcia L, Ellis RE, Peters TM (2003). Comparison of an optical and a mechanical navigation system. Proceedings of Medical Image Computing and Computer-Assisted Intervention – MICCAI 2003, LNCS 2879, 6th International Conference: November 15–18, 2003; Montréal, Canada.

[B12] Martelli S, Zaffagnini S, Bignozzi S, Lopomo N, Marcacci M (2007). Description and validation of a navigation system for intra-operative evaluation of knee laxity. Comput Aided Surg.

[B13] Marcacci M, Molgora AP, Zaffagnini S, Vascellari A, Iacono F, Presti ML (2003). Anatomic double-bundle anterior cruciate ligament reconstruction with hamstrings. Arthroscopy.

[B14] Martelli S (2003). New method for simultaneous anatomical and functional studies of articular joints and its application to the human knee. Comput Methods Programs Biomed.

[B15] McPherson A, Karrholm J, Pinskerova V, Sosna A, Martelli S (2005). Imaging knee position using MRI, RSA/CT and 3D digitization. J Biomech.

[B16] Zaffagnini S, Martelli S, Garcia L, Visani A (2004). Computer analysis of PCL fibres during range of motion. Knee Surg Sports Traumatol Arthrosc.

[B17] Grood ES, Suntay WJ (1983). A joint coordinate system for the clinical description of three-dimensional motions: application to the knee. J Biomech Eng.

[B18] Martelli S, Zaffagnini S, Bignozzi S, Bontempi M, Marcacci M (2006). Validation of a new protocol for computer-assisted evaluation of kinematics of double-bundle ACL reconstrucion. Clinic Biomech (Bristol, Avon).

[B19] Hagemeister N, Long R, Yahia L, Duval N, Krudwig W, Witzel U, de Guise JA (2002). Quantitative comparison of three different types of anterior cruciate ligament reconstrucion methods: laxity and 3-D kinematic measurements. Biomed Mater Eng.

[B20] Noyes FR, Grood ES, Suntay WJ (1989). Three-dimensional motion analysis of clinical stress tests for anterior knee subluxations. Acta Orthop Scand.

[B21] Sakane M, Livesay GA, Fox RJ, Rudy TW, Runco TJ, Woo S (1999). Relative contribution of the ACL, MCL, and bony contact to anteiror stability of the knee. Knee Surg Sports Traumatol Arthrosc.

[B22] Sapega AA, Moyer RA, Schneck C, Komalahiranya N (1990). Testing for isometry during reconstruction of the anterior cruciate ligament. J Bone Joint Surg Am.

[B23] Zelle BA, Vidal AF, Brucker PU, Fu FH (2007). Double-bundle reconstruction of the anterior cruciate ligament: anatomic and biomechanical rationale. J Am Acad Orthop Surg.

[B24] Yasuda K, Kondo E, Ichiyama H, Tanabe Y, Tohyama H (2006). Clinical evaluation of anatomic double-bundle anterior cruciate ligament reconstruction procedure using hamstring tendon grafts: comparisons among 3 different procedures. Arthroscopy.

[B25] Vercillo F, Woo SL, Noorani SY, Dede O (2007). Determination of a safe range of knee flexion angles for fixation of the grafts in double-bundle anterior cruciate ligament reconstruction: a human cadaveric study. Am J Sports Med.

[B26] Mushal V, Burkart A, Debski RE, Van Scyoc A, Fu FH, Woo SL (2003). Anterior cruciate ligament tunnel placement: comparison of insertion site anatomy with the guidelines of a computer-assisted surgical system. Arthroscopy.

